# Supplementary material for: At-home wearables and machine learning sensitively capture disease progression in amyotrophic lateral sclerosis
Source: Nat Commun. 2023 Aug 21;14:5080. doi: 10.1038/s41467-023-40917-3 (PMC10442344; doi:10.1038/s41467-023-40917-3)
Supplement: Supplementary file 1 — Supplementary Information [file 41467_2023_40917_MOESM1_ESM.pdf]

Supplementary Material

Supplementary Table 1. Descriptions for each type of wearable sensor feature.

Feature Level	Feature(s) Name	N	Description
Activity Index	**AI Mean**	1	Activity Index[1] was computed for each 1s window of triaxial accelerometer data over the recording period. Activity intensity (AI) mean is the mean activity index value over all daytime activity over the week-long period. *Periods of inactivity are excluded from the calculation of AI mean, AI median, AI mode, and AI entropy.*[2]
	AI Median	1	Median activity intensity over all daytime activity.
	AI Mode	1	The most common value (mode) of activity intensity over all daytime activity.
	**AI Entropy**	1	The entropy of the distribution of daytime activity intensity.
	% daytime with low AI	1	The percentage of daytime that is spent performing low intensity movements as previously defined.[2]
	% daytime with moderate AI	1	The percentage of daytime that is spent performing moderate intensity movements.
	% daytime with high AI	1	The percentage of daytime that is spent performing high intensity movements.
	% accel in single direction	3	For each one second window of movement, principal component analysis was performed on the triaxial accelerometer data to identify the principal direction of acceleration. This feature is the percentage of accelerometer data variance explained by the first principal component direction, averaged over one second windows. This measure was computed separately for low AI, moderate AI, and high AI one second windows resulting in three features.
Spectral	Total Power	1	Cumulative power in the 0.1-5 Hz frequency band.
Activity Bout	Bout Acceleration	2	"Activity bouts" are continuous periods of activity with durations between 4-18s long based on an activity index threshold.[3] Bout acceleration is the maximum acceleration in m/s^2 during an activity bout. *M and SD are computed over a participant's activity bouts resulting in two features (applies to bout acceleration and bout jerk).*
	Bout Jerk	2	Bout jerk is the mean jerk (derivative of acceleration) in m/s^3 during an activity bout.
SM	**SM Distance**	8	The distance in meters traveled during a submovement (SM). *Mean and standard deviation are computed over a participant's SMs for short duration and long duration SMs in the primary and secondary directions of planar movement resulting in 2*2*2 = 8 total features (applies to SM distance, velocity, acceleration, jerk, and duration).*
	**SM Velocity**	8	The maximum velocity in m/s during a SM.
	**SM Acceleration**	8	The maximum acceleration in m/s^2 during a SM.
	SM Jerk	8	The normalized jerk of a SM. This measure is dimensionless and is scaled based on SM duration and SM peak velocity.[4–6]
	SM Duration	8	The duration of a SM in seconds.
	SM PC1 Score	6	The principal component 1 (PC1) score for a submovement. PC1 captures low-frequency characteristics of the SM velocity-time curve (e.g., the SM "shape"). The PC1 "basis function" is a single sinusoidal waveform with the peak present in the first half of the submovement.[3] *Mean absolute value, standard deviation, and*

			*kurtosis are computed for long duration SMs in the primary and secondary directions of movement resulting in 3*2 = 6 total features (applies to SM PC1-5 scores).*
	SM PC2 Score	6	The principal component 2 score for a submovement. Similar to PC1, PC2 captures low-frequency characteristics of the SM velocity-time curve. The PC2 basis function is a single sinusoidal waveform with the peak present in the second half of the submovement.[3]
	SM PC3-5 Scores	18	The principal component 3-5 scores for a submovement. PC3-5 scores capture higher frequency characteristics of the SM velocity-time curve. The PC3, PC4, and PC5 basis functions consist of 1.5, 2, and 2.5 sinusoidal cycles, respectively.[3]

[a]Bolded features were preselected for individual feature analysis.
[b]Abbreviations: N – Number of features; AI – Activity Intensity; SM – Submovement; PC – Principal Component; M – Mean; SD – Standard Deviation; s – seconds

Supplementary Table 2. Cross-sectional properties of right ankle sensor models for female participants. For relationships with ALSFRS-R and right-left agreement, p-values for Pearson's correlation were computed using a Student's t distribution for a transformation of the correlation (two-tailed test). Mann-Whitney *U*-test was used for ALS versus control comparisons (two-sided test) and Cohen's *d* was used to measure effect size. The Benjamini-Hochberg method was used to adjust for multiple comparisons and corrected p-values are reported.

Sensor	Feature Name	Relationship with ALSFRS-R										Test-retest reliability	ALS vs Control		Right and Left Ankle Agreement	
		Total		Gross Motor		Fine Motor		Respiratory		Bulbar						
		r	p-val	r	p-val	r	p-val	r	p-val	r	p-val	ICC	p-val	es	r	p-val
Ankle (Models)	ALSFRS Total Prediction Model	0.40	6.0E-52	0.55	2.0E-107	0.43	9.0E-63	-	n.s.	-	n.s.	0.88	2.0E-12	0.9	0.88	0
	ALSFRS Gross Motor Prediction Model	0.41	3.0E-54	0.66	4.0E-169	0.42	2.0E-59	-	n.s.	-0.06	3.0E-02	0.92	5.0E-21	1.3	0.94	0
	Pairwise Model	0.46	7.0E-73	0.66	3.0E-170	0.48	2.0E-76	-	n.s.	-	n.s.	0.94	3.0E-15	1.1	0.93	0

Abbreviations: ALSFRS – Amyotrophic Lateral Sclerosis Functional Rating Scale; ICC – Intraclass correlation coefficient; r – Pearson correlation coefficient; es – effect size; n.s. – not significant.

Supplementary Table 3. Cross-sectional properties of right ankle sensor models for male participants. For relationships with ALSFRS-R and right-left agreement, p-values for Pearson's correlation were computed using a Student's t distribution for a transformation of the correlation (two-tailed test). Mann-Whitney *U*-test was used for ALS versus control comparisons (two-sided test) and Cohen's *d* was used to measure effect size. The Benjamini-Hochberg method was used to adjust for multiple comparisons and corrected p-values are reported.

Sensor	Feature Name	Relationship with ALSFRS-R										Test-retest reliability	ALS vs Control		Right and Left Ankle Agreement	
		Total		Gross Motor		Fine Motor		Respiratory		Bulbar						
		r	p-val	r	p-val	r	p-val	r	p-val	r	p-val	ICC	p-val	es	r	p-val
Ankle (Models)	ALSFRS Total Prediction Model	0.66	0	0.71	0	0.55	5.0E-240	0.35	1.0E-87	0.29	2.0E-60	0.88	4.0E-45	1.1	0.93	0
	ALSFRS Gross Motor Prediction Model	0.59	6.0E-280	0.82	0	0.41	4.0E-123	0.30	1.0E-61	0.18	5.0E-24	0.91	2.0E-60	1.4	0.96	0
	Pairwise Model	0.61	3.0E-303	0.79	0	0.43	3.0E-138	0.30	7.0E-65	0.24	7.0E-39	0.92	6.0E-61	1.4	0.94	0

Abbreviations: ALSFRS – Amyotrophic Lateral Sclerosis Functional Rating Scale; ICC – Intraclass correlation coefficient; r – Pearson correlation coefficient; es – effect size.

Supplementary Table 4. Cross-sectional properties of right wrist sensor models for female participants. For relationships with ALSFRS-R and right-left agreement, p-values for Pearson's correlation were computed using a Student's t distribution for a transformation of the correlation (two-tailed test). Mann-Whitney *U*-test was used for ALS versus control comparisons (two-sided test) and Cohen's *d* was used to measure effect size. The Benjamini-Hochberg method was used to adjust for multiple comparisons and corrected p-values are reported.

Sensor	Feature Name	Relationship with ALSFRS-R										Test-retest reliability	ALS vs Control		Right and Left Wrist Agreement	
		Total		Gross Motor		Fine Motor		Respiratory		Bulbar						
		r	p-val	r	p-val	r	p-val	r	p-val	r	p-val	ICC	p-val	es	r	p-val
Wrist (Models)	ALSFRS Total Prediction Model	0.40	9.0E-52	0.31	7.0E-32	0.48	2.0E-77	-	n.s.	0.16	3.0E-09	0.84	5.0E-14	1.0	0.81	0
	ALSFRS Fine Motor Prediction Model	0.37	2.0E-45	0.29	2.0E-26	0.53	7.0E-99	-	n.s.	0.10	9.0E-04	0.83	8.0E-16	1.0	0.77	2.6E-278
	Pairwise Model	0.38	2.0E-48	0.26	2.0E-22	0.49	4.0E-82	-	n.s.	0.18	1.0E-10	0.90	5.0E-21	1.2	0.86	0

Abbreviations: ALSFRS – Amyotrophic Lateral Sclerosis Functional Rating Scale; ICC – Intraclass correlation coefficient; r – Pearson correlation coefficient; es – effect size; n.s. – not significant.

Supplementary Table 5. Cross-sectional properties of right wrist sensor models for male participants. For relationships with ALSFRS-R and right-left agreement, p-values for Pearson's correlation were computed using a Student's t distribution for a transformation of the correlation (two-tailed test). Mann-Whitney *U*-test was used for ALS versus control comparisons (two-sided test) and Cohen's *d* was used to measure effect size. The Benjamini-Hochberg method was used to adjust for multiple comparisons and corrected p-values are reported.

Sensor	Feature Name	Relationship with ALSFRS-R										Test-retest reliability	ALS vs Control		Right and Left Wrist Agreement	
		Total		Gross Motor		Fine Motor		Respiratory		Bulbar						
		r	p-val	r	p-val	r	p-val	r	p-val	r	p-val	ICC	p-val	es	r	p-val
Wrist (Models)	ALSFRS Total Prediction Model	0.69	0	0.64	0	0.70	0	0.35	7.0E-86	0.32	4.0E-72	0.84	7.0E-76	1.6	0.87	0
	ALSFRS Fine Motor Prediction Model	0.68	0	0.55	3.0E-239	0.75	0	0.32	2.0E-73	0.33	1.0E-75	0.85	2.0E-73	1.5	0.83	0
	Pairwise Model	0.65	0	0.54	4.0E-222	0.70	0	0.27	7.0E-50	0.37	3.0E-96	0.90	4.0E-98	1.9	0.85	0

Abbreviations: ALSFRS – Amyotrophic Lateral Sclerosis Functional Rating Scale; ICC – Intraclass correlation coefficient; r – Pearson correlation coefficient; es – effect size.

Supplementary References

1. Bai J, Di C, Xiao L, et al. An activity index for raw accelerometry data and its comparison with other activity metrics. *PLoS One*. 2016;11(8):1-14.

2. Khan NC, Pandey V, Gajos KZ, Gupta AS. Free-Living Motor Activity Monitoring in Ataxia-Telangiectasia. *Cerebellum*. 2021;(0123456789). doi:10.1007/s12311-021-01306-y

3. Gupta AS, Luddy AC, Khan NC, Reiling S, Thornton JK. Real-life Wrist Movement Patterns Capture Motor Impairment in Individuals with Ataxia-Telangiectasia. *Cerebellum*. 2022;(0123456789). doi:10.1007/s12311-022-01385-5

4. Balasubramanian S, Melendez-Calderon A, Burdet E. A robust and sensitive metric for quantifying movement smoothness. *IEEE Transactions on Biomedical Engineering*. 2012;59(8):2126-2136.

5. Hogan N, Sternad D. Sensitivity of smoothness measures to movement duration, amplitude, and arrests. *J Mot Behav*. 2009;41(6):529-534.

6. Gajos KZ, Reinecke K, Donovan M, et al. Computer Mouse Use Captures Ataxia and Parkinsonism, Enabling Accurate Measurement and Detection. *Movement Disorders*. 2020;35(2):354-358. doi:10.1002/mds.27915
