# Supplementary material for: At-home wearables and machine learning sensitively capture disease progression in amyotrophic lateral sclerosis
Source: Nat Commun. 2023 Aug 21;14:5080. doi: 10.1038/s41467-023-40917-3 (PMC10442344; doi:10.1038/s41467-023-40917-3)
Supplement: Supplementary file 3 — Reporting Summary [file 41467_2023_40917_MOESM3_ESM.pdf]

nature portfolio

Corresponding author(s): Anoopum Gupta

Last updated by author(s): Aug 3, 2023

Reporting Summary

Nature Portfolio wishes to improve the reproducibility of the work that we publish. This form provides structure for consistency and transparency in reporting. For further information on Nature Portfolio policies, see our Editorial Policies and the Editorial Policy Checklist.

Statistics

For all statistical analyses, confirm that the following items are present in the figure legend, table legend, main text, or Methods section.

n/a	Confirmed	
☐	☒	The exact sample size (n) for each experimental group/condition, given as a discrete number and unit of measurement
☐	☒	A statement on whether measurements were taken from distinct samples or whether the same sample was measured repeatedly
☐	☒	The statistical test(s) used AND whether they are one- or two-sided<br>*Only common tests should be described solely by name; describe more complex techniques in the Methods section.*
☐	☒	A description of all covariates tested
☐	☒	A description of any assumptions or corrections, such as tests of normality and adjustment for multiple comparisons
☐	☒	A full description of the statistical parameters including central tendency (e.g. means) or other basic estimates (e.g. regression coefficient) AND variation (e.g. standard deviation) or associated estimates of uncertainty (e.g. confidence intervals)
☐	☒	For null hypothesis testing, the test statistic (e.g. F, t, r) with confidence intervals, effect sizes, degrees of freedom and P value noted<br>*Give P values as exact values whenever suitable.*
☒	☐	For Bayesian analysis, information on the choice of priors and Markov chain Monte Carlo settings
☒	☐	For hierarchical and complex designs, identification of the appropriate level for tests and full reporting of outcomes
☐	☒	Estimates of effect sizes (e.g. Cohen's d, Pearson's r), indicating how they were calculated

Our web collection on statistics for biologists contains articles on many of the points above.

Software and code

Policy information about availability of computer code

Data collection	No software was used for data collection.
Data analysis	Statistical analyses were completed in MATLAB version R2022a (Mathworks, Natick, MA).

For manuscripts utilizing custom algorithms or software that are central to the research but not yet described in published literature, software must be made available to editors and reviewers. We strongly encourage code deposition in a community repository (e.g. GitHub). See the Nature Portfolio guidelines for submitting code & software for further information.

Data

Policy information about availability of data

All manuscripts must include a data availability statement. This statement should provide the following information, where applicable:

- Accession codes, unique identifiers, or web links for publicly available datasets
- A description of any restrictions on data availability
- For clinical datasets or third party data, please ensure that the statement adheres to our policy

The GTX3 accelerometer data and associated ALSFRS-R data are available upon request because file sizes necessitate coordinated data transfer. Access can be obtained by visiting https://www.als.net/arc/data-commons/ and requesting the dataset by submitting accession code 06162023.

Field-specific reporting

Please select the one below that is the best fit for your research. If you are not sure, read the appropriate sections before making your selection.

☒ Life sciences ☐ Behavioural & social sciences ☐ Ecological, evolutionary & environmental sciences

For a reference copy of the document with all sections, see nature.com/documents/nr-reporting-summary-flat.pdf

Life sciences study design

All studies must disclose on these points even when the disclosure is negative.

Sample size	The natural history study which produced the wearable sensor data for this manuscript had multiple goals and aimed to recruit 300 ALS participants in Phase I and up to 700 ALS participants in Phase 2. These sample sizes were based on rough estimates for the number of cell lines and genomic sequences needed for genotype-phenotype analyses in ALS as well as the number of samples needed to detect small changes in ALSFRS-R progression slope. A sample size was not predetermined for the accelerometer-based analyses, however all comparisons performed in the study were highly significant, and the sample size was very large in comparison to other observational studies in ALS and in comparison with our prior submovement-based wearable sensor studies in cerebellar ataxias.
Data exclusions	The exclusion criteria used in this study were determined prior to initiating data analysis, but not prior to data collection. As described below, the results were robust to other criteria. The data filtering steps are described in detail in the manuscript: "The dataset filtering steps are described in Figure 1B. Cross sectional analysis included 4637 sessions from 402 unique participants (376 ALS, 26 controls) with at least 24 hours of recorded accelerometer data, pooled only from days with at least 3 hours of data, from all four limbs (Figure 1B). The 24 hour session minimum for daytime data was chosen based on prior work demonstrating high reliability of daytime data across the first three and last three days in a week20,21. Longitudinal analysis was conducted using data from participants with at least three data collection sessions spanning a minimum of 0.75 years (188 ALS and 6 control participants). "
Replication	All results presented in the manuscript were reproducible. The longitudinal analysis was replicated three times using other criteria (i.e., minimum of 0.5, 1, and 1.5 years of longitudinal data) and the results were qualitatively unchanged.
Randomization	There was no allocation into groups in this observational study.
Blinding	There was no allocation into groups in this observational study.

Reporting for specific materials, systems and methods

We require information from authors about some types of materials, experimental systems and methods used in many studies. Here, indicate whether each material, system or method listed is relevant to your study. If you are not sure if a list item applies to your research, read the appropriate section before selecting a response.

Materials & experimental systems

n/a	Involved in the study	
☒	☐	Antibodies
☒	☐	Eukaryotic cell lines
☒	☐	Palaeontology and archaeology
☒	☐	Animals and other organisms
☐	☒	Human research participants
☒	☐	Clinical data
☒	☐	Dual use research of concern

Methods

n/a	Involved in the study	
☒	☐	ChIP-seq
☒	☐	Flow cytometry
☒	☐	MRI-based neuroimaging

Human research participants

Policy information about studies involving human research participants

Population characteristics	ALS participants ranged in age from 21-79 with a median age of 57.
Recruitment	Emails were sent to ALS TDI's mailing list describing the program following IRB approval. The email provided a description of the procedures, the aims of the study, a description of the required consent and waivers, and contact information. All recruitment emails link to a page on the ALS TDI website (www.als.net) describing the study and an opportunity to submit inquiries to participate. The possibility of self-selection bias arises in this study, as participants were volunteers rather than being randomly chosen. It's likely that individuals who can afford the time to partake in natural history studies are of a higher socioeconomic stratum, rendering the sample less diverse and potentially not fully reflective of the broader population.
Ethics oversight	This research study was conducted in accordance with the ethical principles posited in the Declaration of Helsinki - Ethical Principles for Medical Research Involving Human Subjects. Protocol approval was provided by the institutional review board

(ADVARRA CIRBI). Every participant consented to participate in this research by signing an IRB approved informed consent form. There was no participant compensation in this study.

Note that full information on the approval of the study protocol must also be provided in the manuscript.
